# Intensive Livestock Farming and Residential Health: Experts’ Views

**DOI:** 10.3390/ijerph16193625

**Published:** 2019-09-27

**Authors:** Valérie Eijrond, Liesbeth Claassen, Joke van der Giessen, Danielle Timmermans

**Affiliations:** 1 Amsterdam UMC, Vrije Universiteit Amsterdam, Department of Public and Occupational Health, Amsterdam Public Health research institute, Van der Boechorststraat 7, NL-1081 BT Amsterdam, The Netherlands; liesbeth.claassen@rivm.nl (L.C.); drm.timmermans@amsterdamumc.nl (D.T.); 2 Centre for Environmental Security and Safety, National Institute for Public Health and the Environment (RIVM), 3720 BA Bilthoven, The Netherlands; 3 Centre for Infectious Disease Control Netherlands, National Institute for Public Health and the Environment (RIVM), 3720 BA Bilthoven, The Netherlands; joke.van.der.giessen@rivm.nl

**Keywords:** intensive livestock farming, mental models

## Abstract

The presence of intensive livestock farms in close vicinity to residential areas in the Netherlands is a complex problem characterised by knowledge uncertainty about the effects on residential health, overlapping value-driven concerns and stakeholder diversity. In order to address concerns about the health effects and effectively manage the debate about intensive livestock farming, constructive stakeholder dialogues are encouraged, informed by current scientific insights. We explored the current knowledge, beliefs and concerns of scientific experts, following the mental models approach. A summary expert model was derived from scanning the relevant literature and informed by interviews with 20 scientific experts. The study shows imprecise use of terminology by experts. Moreover, they appear to perceive intensive livestock farming not as a major health problem at least at this moment for neighbouring residents in the Netherlands. Broader themes such as (environmental) unsustainability and biodiversity loss seem a more prominent concern among the experts. Our study questions whether dialogues should only focus on residential health or cover broader values and concerns. However, mental models about risk may differ with other stakeholders, impeding communication. Hence, we will identify other stakeholders’ knowledge, beliefs and value-based concerns in the light of facilitating constructed dialogues between stakeholders.

## 1. Introduction

Currently, there is a growing societal debate in the Netherlands about the future of intensive livestock farming. The Netherlands is a densely populated country with a high people to livestock ratio: on a surface area of approximately 41.500 km^2^, 17 million people live together with circa 100 million broilers and laying hens, 12 million pigs, 4 million cows, 0.8 million sheep and 0.5 million goats [1]. In response to the food shortage after the World Wars, the livestock industry has faced scale enlargement and intensification [2,3]. Lately, there has been a shift in the post-war values of producing sufficient, affordable food and income from exports to sustainable and ethical values such as animal welfare and the environment [2,3]. Moreover, there is discussion about the potential human health risks specifically for residents of nearby communities, predominantly in two Dutch provinces with the highest density of livestock farms in the Netherlands: Noord-Brabant and Limburg. This discussion has accelerated since the Netherlands experienced the world’s largest Q fever outbreak in 2007–2010 associated with intensive dairy goat farming, with 4000 notified human cases, of which approximately 74 were fatalities [4]. The Q fever outbreak in a high livestock and resident-dense region in the Netherlands triggered the demand for more knowledge about health effects for residents living in close proximity of livestock farms [5].

While the occupational health implications of livestock are known, little is known about the health risks of livestock farming for neighbouring residents [2]. The human health hazards of intensive livestock farming for the surrounding, non-farming population is bound up to knowledge uncertainty. Epidemiological associations have been found, yet the causal relationships remain unclear [5,6,7,8]. A large-scale livestock farming and health (VGO) study (Acronym for Veehouderij en Gezondheid [ = Livestock Farming Neighbouring Residents’ Health]) found an association between pneumonia and living within 1 km of poultry farms (between 2009–2014) and 2 km of goat farms (between 2009–2016). An association was also found between reduced lung function and living within 1 km of 15 or more livestock farms and with high ammonia concentrations. In addition, people living within these distances with Chronic Obstructive Pulmonary Disease (COPD) experienced exacerbations (worsening of the symptoms). However, the study also suggests that living near livestock farms have protective effects: asthma, allergies and COPD occur less frequently in persons in proximity to livestock farms. Moreover, research into some specific zoonotic pathogens and antibiotic resistance microorganisms showed no significant associations with living near livestock farms [5].

Apart from residential health, there are many other overlapping value-driven concerns contributing to the complexity of intensive livestock farming. Issues entering the social and political agenda range from environmental pollution, to biodiversity loss, animal welfare concerns, landscape impairment, cultural history loss and other human health hazards such as food safety and antibiotic resistance [4,5]. In the face of these value-driven concerns, there is a growing collective request for a more sustainable livestock production system. However, little consensus exists regarding the potential solutions as intensive livestock farming is an interdependent system and there are trade-offs among competing concerns. Addressing one domain of the problem may translate into a new problem or aggravate an existing problem within another concern. For instance, placing a large industrial farm with a closed system on an industrial area limits public health risks, but may lead to concerns for those advocating for animal welfare. Small-scale or extensive animal husbandry may stimulate the natural behaviour of animals but presents a greater risk for the spread of zoonotic pathogens such as avian influenza from wild birds to free ranging poultry, and possibly to residents living in close proximity.

Additionally, there is considerable stakeholder diversity. A large number of stakeholders are involved in the health debate. These include farmers, local residents, interest groups, general practitioners/medical specialists, scientists, government officials and many more [9]. A multitude of actors implies that there is a diversity of “knowledge pools, public values and social interests” [10] that need to be considered in the debate. If these are dismissed or misunderstood in stakeholder interactions, stakeholders may feel disregarded, possibly resulting in conflicts and polarisation.

The high complexity, which includes high levels of knowledge uncertainty and interdependencies as well as high stakeholder diversity [11], implies that intensive livestock farming can be viewed as a ‘very wicked problem’, a term coined by Rittel and Webber [12], along with global climate change, poverty and genetically-modified food [13,14]. This entails that technical solutions and administrative solutions (top-down) used to solve less-complex and less-diverse problems are insufficient in resolving wicked problems “with their attendant conflict over social values and high uncertainty about system components and outcomes” [15,16]. Some form of collaboration or facilitated dialogue is advocated to deal with wicked problems as it increases the likelihood that the problem is understood, solutions can be found and agreed upon, and facilitates the implementation of the solutions. These conditions can be fulfilled when collaborations tap a wide range of experts as well as situational knowledge and skills [16,17]. Nevertheless, collaborations are difficult to establish and require transparent communication, trust and mutual commitment.

Scientific experts have a particular type of knowledge authority and are often called upon to inform these collaborations by providing their perspective on the current scientific findings. To help improve this dialogue, we propose to employ a mental models approach [18]. In the mental models approach, experts’ knowledge and insights of a risk are compared with the understanding of potential audiences to identify relevant differences that can inform the content of communications. In this paper, we assess the scientific experts’ perspective on human health and intensive livestock farming to complete the first phase of a mental models approach [18]. The aim of this paper is threefold. First, to explore the current scientific knowledge and insights about the relationship between intensive livestock farming and human health for neighbouring residents. To start, we scanned the (inter)national literature to identify the potential hazards associated with living near intensive livestock farms as well as the possible associated health effects. Second, to place the hazards and health effects into context and perspective: to judge whether it is a major problem for residents in the Netherlands by interviewing scientific experts. Third, the aim of the interviews is also to explore experts’ broader concerns, as under conditions of high uncertainty, experts tend to express their views beyond their area of expertise [19].

## 2. Materials and Methods

### 2.1. Design

Following the mental models approach [18], we systematically explore and provide an aggregated and comprehensive overview, expert model, capturing the current scientific knowledge, beliefs and concerns regarding residential health and intensive livestock farming. In order to create a so-called expert model, first information was derived from scanning the relevant literature: information from peer-reviewed articles as well as grey literature including government publications by using English and Dutch search terms such as intensive livestock farming, human health, residents and the Netherlands. We also conducted backward searching and consulted experts to check whether the relevant literature was not overlooked. Subsequently, face-to-face interviews were conducted with experts to place the hazards and health effects found in the literature into context and perspective.

### 2.2. Sample Expert Selection

There is no fixed definition of what constitutes an expert [19]. In this study, we considered experts as scientists, who have at least a PhD and whose past or present fields of expertise included scientific research and publications in peer-reviewed journals into various aspects of livestock farming in the Netherlands (Table 1). Some experts are academic researchers, researchers at research institutes and/or advisors. The sample of scientific experts was identified and selected according to the literature reviewed as well as suggestions given by the advisors of this study. Since the study was interested in the human health hazards of intensive livestock farming, care was taken to select scientific experts with different backgrounds, including public health (*n* = 5) and veterinary sciences (*n* = 5) and areas of expertise within other domains such as engineering, toxicology and molecular biology (*n* = 5). Their expertise encompassed infectious diseases caused by (pathogenic) microorganisms and environmental pollutants such as odour, particulate matter and endotoxins. As intensive livestock farming is a social issue, cross-cutting multiple value-driven domains, social scientists (*n* = 5) with specific expertise in the field of livestock farming were also interviewed. Prior to the interviews, informed consent was obtained including requesting permission to audio record the interviews.

### 2.3. Procedure

Interview guidelines were developed and verified among four project advisors. The selected experts were invited by email to participate. All invited experts agreed to participate. Semi-structured interviews were held between January and March 2018 with 20 scientific experts in the Netherlands. According to Morgan et al. [18], saturation usually occurs after 10–12 interviews. These face-to-face interviews were carried out by a single interviewer and lasted on average 60 minutes. In order to elicit the scientific expert perspectives, the interviews commenced with a question about their professional work relation with intensive livestock farming. Subsequently, interviewees were asked to share their interpretation of the term intensive livestock farming. This was followed by knowledge-specific questions on human health and intensive livestock farming. These questions included: “Could you tell me what human health has to do with intensive livestock farming” and “What are the human health risks of intensive livestock farming”? These broadly formulated questions refrain the interviewer “leading the witness” and the risk of missing important beliefs [18]. Next, a list of human health hazards associated with intensive livestock farming identified by the researcher based on the literature was presented. The respondents were asked to comment on this list. Afterwards, belief-specific questions were asked, whereby scientific experts were asked to rank this list (printed in cards of A6 format) according to what they consider to be the largest and smallest human health problem in relation to intensive livestock farming. This ranking task aimed to uncover additional beliefs not found earlier in the interview. Subsequently, a question was asked regarding what other themes they associated with intensive livestock farming. Towards the end of the interview, the interviewer asked whether, as a scientist, they were concerned about intensive livestock farming as well as what they supposed citizens were concerned about. Lastly, experts were asked, at the end of the interview, to suggest potential additional respondents to interview to ensure that relevant scientific experts in the field were included in the study. See the Supplementary Materials for the full interview protocol.

### 2.4. Coding

All interviews were audio recorded and transcribed. These were analysed by one researcher (VE) through open coding using ATLAS.ti, allowing the identification of common themes. A second researcher (LC) independently coded two interviews in order to ensure that all relevant themes were identified. Based on the analysis of the interviews and literature, a draft expert model was constructed, including an explanation. This was sent to all interviewees where they were asked to review the model from their area of expertise. Their comments were used to finalise the model.

## 3. Results

**Figure 1:**
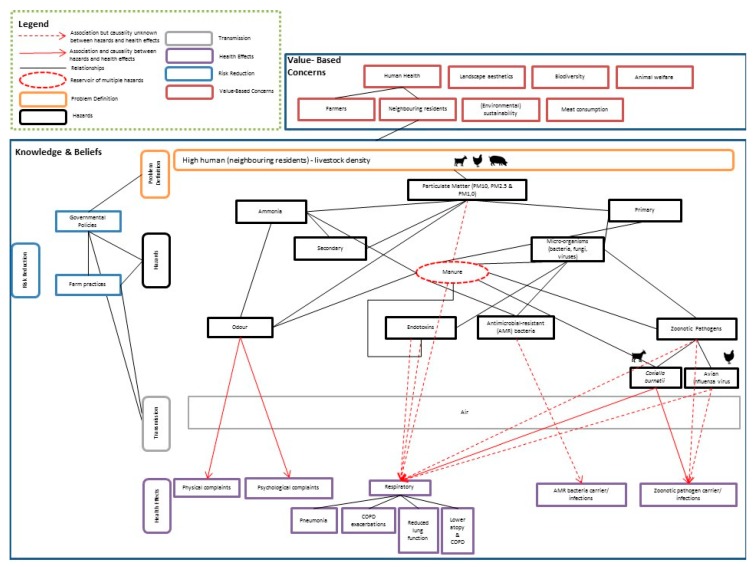
Final expert model of scientific experts on residential health and intensive livestock farming.

Based on the literature and the results of the expert elicitations, an expert model was designed as shown in Figure 1. The model described the combined knowledge, beliefs and concerns of scientific experts regarding the relationship between human health and intensive livestock farming for residents living in the vicinity of livestock farms. The expert model consists of six coloured parts: problem definition (orange), human health hazards (black), transmission (grey), risk reduction (blue), health effects (purple) and value-based concerns (red).

### 3.1. Problem Definition

Much of the societal debate in the Netherlands about the health risks is framed around the term “intensive livestock farming”. However, as interpreted from the expert interviews, this term does not fully capture the problem. Moreover, there is a lack of consensus about the definition. The Dutch Council of State [20] defines it as an agricultural business keeping livestock, which include cattle, pigs, veal, poultry, minks, goats or sheep or a combination of these, with the exception of land-based dairy cattle farming. In other words, agricultural businesses where farmers do not have (sufficient) land to grow feed and hence purchase feed for their livestock.

Although some experts provided a similar definition, most experts associated intensive livestock farming with poultry, pigs and to a lesser extent goat farms. Throughout the interviews the focus was also on the health hazards originating from these three livestock types. Other animal species such as beef cattle, minks and sheep were not mentioned. Dairy cattle, which is not defined as intensive livestock but is known to emit high amounts of ammonia, hence contributing to secondary particulate matter and odour (see Section 3.2), was only mentioned by a few experts. Most experts associated intensive livestock farming with the number of animals and scale: many animals (without quantifying) concentrated on a small surface area. Whether this refers to many animals situated close together or many animals situated in densely human populated areas is unclear.

As interpreted from the interviews, the main problem according to the experts is the high human –livestock density. The distance between livestock and residents is declining, a gradual trend that has taken place over the past decade. As a result, those living in close proximity of livestock farms are exposed to potential health hazards. Current scientific evidence illustrates associations according to the distance between farms and neighbouring residents [5,21]. According to the literature there is no evidence linking the scale of an individual farm to health risk; no associations between large-scale individual farms, so-called “mega farms” [22,23] and health effects were found [21,24]. This is confirmed by the interview results as the experts point out that large farms provide opportunities for technical innovations that actually have less health risks (i.e., advanced systems to control emissions) in comparison with older stables even where the number of animals might be lower. Furthermore, they state that extensive farms, described as biological farms, small scale farms or farms where animals can roam freely outdoors, may pose a greater health risk. Many clarify this by using poultry and avian influenza as an example. Poultry that roam freely outdoors are easily exposed to pathogens such as avian influenza that are transmitted from wild birds. When poultry is kept indoors, they are less likely or unexposed to pathogens from wild birds, and hence, to avian influenza.

### 3.2. Human Health Hazards

Livestock farms emit various compounds into the atmosphere. As shown in Figure 1, the following key hazards were identified based on the literature and expert interviews (see the Supplementary Materials for further explanation on the relationships and associations): particulate matter (PM), primary and secondary PM, ammonia, microorganisms, endotoxins, antimicrobial resistant bacteria, zoonotic pathogens (*Coxiella burnetii* and avian influenza) and odour.

When experts were asked about human health in relation to intensive livestock farming, frequent references were made to the following health hazards: particulate matter, antibiotic-resistant bacteria (specifically Methicillin Resistant Staphylococcus aureus—MRSA), odour, zoonotic diseases and ammonia. Two zoonotic pathogens were frequently mentioned: avian influenza virus found in poultry and *Coxiella burnetii* present in goats. Most experts mentioned manure as a problem, which consists of animal faeces used as a fertilizer in agriculture. In this model, it is considered a reservoir of various hazards mostly in the context of health by emitting odour and microorganisms. Some also pointed out the issue of manure fraud as well as the negative effects on the environment. Five experts also mentioned fungi, yet stated that the associations between livestock farming and residential health is unclear due to a lack of research. Other possible hazards identified in the literature, yet seldom brought up by the experts, include specific zoonotic pathogens such as *Chlamydia psittaci*, *Salmonella spp*, *Campylobacter spp*, Hepatitis E virus; antimicrobial resistant bacteria such as *Clostridium Difficile* and Extended Spectrum Beta-Lactamase producing bacteria (ESBL); and endotoxins.

### 3.3. Transmission

Microorganisms may be transmitted from animals to humans via multiple routes, such as direct contact (skin wounds, mucous membranes or faecal-oral) ingestion of contaminated food, water and by air [25]. Air is also a transmission route for primary and secondary PM. Since the focus of the study is on the health hazards for neighbouring residents, air is considered the main transmission route that is considered a risk for people living in close vicinity of livestock farms. The transmission of health hazards through water and food is a risk factor for the general population, not restricted to living near livestock farms. Direct transmission is limited to farm-related occupations such as farmer and family members, farm personnel and veterinarians. In the expert model, we therefore focus on particles that may (also) be transmitted via air.

### 3.4. Health Effects

Multiple, fairly recent systematic literature reviews summarised the health effects from intensive livestock farming [26,27,28,29]. The uncertain health effects connecting the health hazards are shown by the dotted red lines in Figure 1. More details obtained from the literature can be found in the Supplementary Materials.

#### 3.4.1. Physical and Psychological Complaints Related to Odour

Odours from livestock farms are commonly described by the experts as a form of hindrance, yet studies have shown that odour may also evoke physical and psychological complaints [30,31,32,33,34,35]. Most experts state odour is a large problem, according to what they hear from people living in the vicinity of livestock farms. However, at the same time, they also believe that it does not have a disease burden or evoke physical health effects. In the ranking, odour is often considered a smaller problem compared to other hazards such as PM, known to cause illness and increased mortality. On the other hand, many also acknowledge that odour may have a significant negative impact on people’s wellbeing, cause stress, have psychological health consequences, create irritation and limit people’s daily activities.

“I think that people in the area experience this [odour] as the biggest problem but that this [particulate matter] is probably the biggest problem … When it comes to the impact on public health … I think that people do not get sick of smell and annoyance, probably stress.”

(Expert 3)

“You won’t get sick from smell. It will affect you mentally but not physically. It is also very annoying but it is not deadly.”

(Expert 17)

Also, many experts believe that the public, erroneously, associate odour with other health hazards as shown in the quote below:

“I smell something so apparently that air spreads over my house and over my garden. I smell it and people have also become ill. What else is in it? I think that is what worries people.”

(Expert 16)

#### 3.4.2. Respiratory Effects

The national and international literature reviews state that intensive livestock farming may compromise respiratory health [26,27]. However, the exact nature of the hazards causing these health effects remain unknown. Various zoonotic pathogens, particulate matter and endotoxins may cause respiratory problems but almost all respondents consider PM a large problem. Many experts state that exposure to PM in general may have adverse health effects, causing illness and can be lethal. Furthermore, the general population, hence not necessarily residents, are continually exposed to PM. Also, PM is imperceptible as opposed to odour. With regard to livestock farming, some experts refer to the recent study (VGO) which found associations between living near livestock farms and respiratory effects [5]. However, they do state that the exact causes remain unknown.

“I think that it is a major problem in the Netherlands at all levels: traffic but also with livestock farming. Yet we do not know how large the contribution of livestock farming is to particulate matter. Nonetheless, particulate matter causes a lot of illness and deaths, that is clear.”

(Expert 13)

#### 3.4.3. Antimicrobial Resistant Bacteria Carrier/Infections

Another possible health effect includes residents being carriers or becoming infected with antimicrobial-resistant (AMR) bacteria. The interviewed experts consider AMR a problem for the general population but not necessarily for those living near livestock farms. According to most experts, it is mainly a human-to-human transmission problem. They state there are many measures put in place to reduce the potential risk from livestock e.g., reduction in antibiotic usage in livestock.

#### 3.4.4. Zoonotic Pathogen Carrier/Infections

Zoonotic pathogens such as Hepatitis E virus, *Campylobacter spp*, *Salmonella spp*, *Chlamydia psittaci*, avian influenza virus and *Coxiella burnetii* are potential hazards for neighbouring residents. Most experts mentioned avian influenza and Q fever (the diseases caused by the avian influenza virus and the *Coxiella burnetii bacterium*). Experts consider zoonotic pathogens a potentially large problem due to the risk of unexpected future outbreaks of known or unknown zoonotic pathogens. Especially avian influenza is considered a possible future threat due to potential disruptiveness for humans in terms of its virulence potential as the virus has the capacity to genetically modify and also impact on the poultry sector. Although experts do not consider Q fever a large problem anymore in the Netherlands since there is a mandatory vaccination programme for dairy goats and sheep, they still believe it is a zoonotic pathogen to be vigilant about. Not only because the previous outbreak occurred unexpectedly but also because there are incidental cases of acute Q fever as the bacterium may also be present in other animal species, and there are many people with chronic Q fever as a result of the epidemic. They also point out that neighbouring residents continue to be concerned about Q fever and zoonoses in general. Nonetheless, currently experts do not consider zoonotic pathogens a large problem as there is no immediate threat and there are various monitoring and control programmes on livestock farms.

### 3.5. Risk Reduction

To prevent or reduce the exposure to the problem, hazards and transmission to residents, various measures can be adopted. In the model, these have been differentiated into two categories: governmental policies and farm practices.

Governmental policies include measures to reduce the increase in livestock in the Netherlands. For instance, eight provinces have stopped issuing permits for goat farms [36]. Another measure is increasing the distance between residents and livestock, a criterion taking into account when granting a permit for building or expanding a farm. In the interviews, many experts suggested the industrialisation of livestock farms by moving farms to a remote industrial terrain, far away from densely-populated areas. Furthermore, there are various hygiene policies in the entire food chain such as food safety measures, notification obligations and monitoring programmes (i.e., tank milk checks and vaccination for Q fever, Salmonella monitoring programme and blood testing for avian influenza) to prevent, early detect or eliminate pathogens present in farms and block its transmission. Lastly, there are emission norms for ammonia emissions (law on ammonia and livestock) as well as odour emissions (law on odour nuisance and livestock). The latter law is currently being re-evaluated since research has shown that more odour nuisance is reported than expected based on the current norms [37]. Several experts mention the governments’ goal regarding the reduction of antibiotic usage in livestock. Since 2009, total sales of antibiotics have decreased by 63% [38]

The second category covers measures directed to farm practices. During the interviews, many experts refer to technical innovations of stables. They mentioned the implementation of air filtering systems (chemical, biological and combi) to limit emissions to the environment. Yet, a recent study has shown that combi air filtering systems are significantly less efficient in removing odour and ammonia than expected [39,40]. In addition, experts mention farm hygiene focusing on livestock to prevent infections or transmission. A few experts mention government policies such as animal culling for instance during an avian influenza outbreak, vaccination of goats to prevent *Coxiella burnetii* infections and manure controls. Other possible measures include keeping animals indoors, treating infected animals and separating healthy and sick animals.

### 3.6. Broader Expert Concerns

When experts were asked about their concerns regarding intensive livestock farming, many were fairly concise, stating that they are not concerned about (human health in the context of) intensive livestock farming. More than half of the experts interviewed emphasised that large-scale farms actually contain fewer human health risks compared to small-scale/extensive livestock farms due to the technical innovations of farms which limit the exposure to health hazards. They point out that intensive livestock farms are situated in modern buildings with opportunities to adopt innovative technologies such as ventilation systems to control emissions.

“Zoonoses can also come from one animal and there is of course a lot of debate at this moment about poultry where you sometimes have to deal with bird flu, where the risks of small-scale poultry farms may be greater than for very large intensive farms. These often have better biosecurity compared to smaller farms.”

(Expert 1)

“If you look purely at the health technical aspects and the animal welfare aspects, they are better off in the current livestock farming systems than in the old livestock farming systems.”

(Expert 10)

Nonetheless, when probing further, the majority indicated being concerned about broader aspects, including mainly (environmental) unsustainability and the loss of biodiversity. Some also mentioned the negative effect on landscape aesthetics and the health hazards for farmers.

“Sustainability, the environment, the manure fraud and every time in the news that we produce far too much manure and have far too many animals. The landscape, the nature, the lapwings [type of bird] that are killed by mowing machines, the predatory birds and the insects that are scarcely there, and bird species that are dying out.”

(Expert 5)

“The countryside is not becoming more beautiful. Livestock farming is no longer a small farmer with some animals with a landscape function, but it is increasingly becoming an industry and it is becoming a problem to fit in the landscape. And at some point you can ask why for God’s sake it has to take place in the countryside if it is not land-bound.”

(Expert 7)

Animal welfare is a dominant and recurring theme that experts appear to value but are not necessarily concerned about in relation to intensive livestock farming or large farms. The majority of the interviewees question whether animal welfare is less considered in intensive compared to extensive livestock farms. Many argue that large-scale farms are situated in modern barns, taking animal welfare into consideration.

“… I forgot about animal suffering. Perhaps we should be neater and friendlier… I do not mind that animals are raised for consumption but it has to be done in a decent way.”

(Expert 2)

“There are certainly sustainable and animal welfare friendly large-scale farms. Perhaps especially large-scale farms as it provides economic opportunities to generate a number of conditions in barns in terms of improved air quality, animal welfare and sustainability. You often have to make large investments.”

(Expert 12)

Despite having concerns, most experts did not have a particular negative viewpoint towards intensive livestock farming. They state that it has acquired, too short-sightedly, a negative image within society. In addition, some state that the public has a distorted romantic image of livestock farming in the earlier days.

“Those small farmers do not have air filtering system… therefore the entire business management is much more moderate than the rest, but people get a more romantic image of that. A small peasant farmer in the landscape is more attractive and more fun but whether it is efficient and healthy is very much the question.“

(Expert 13)

However, many experts also believe that livestock farming in the Netherlands has become unmanageable in the long-run. They consider the current livestock farming approach an unsustainable system. Many argue that transformation is necessary and inevitable.

“But now in the Netherlands we have 54 million animals, 4 times as much as the number of inhabitants that is of course completely unmanageable. There is a misbalance.”

(Expert 17)

“It is very crazy that on such a small area we have millions of pigs, millions of cows, have to import food to feed the system and then have to throw all of that manure on our own land. So there is over-fertilization, there is also eutrophication resulting in manure in surface water. This leads to all kinds of problems in ditches and rivers etc. and we have been able to manage it to a certain extent, but it is a huge burden from the livestock industry, which is not sustainable in the long-run.”

(Expert 7)

Many mention that it involves trade-offs between the various value-driven domains.

“… the interests between animal welfare, environmentally friendly, cost price, food safety; those are interests that all demand a different solution… yet we all want it to be safe for the environment, food is safe, welfare of animals is guaranteed and that a farmer can earn a living.”

(Expert 18)

Also, almost half of the experts shared their personal view that meat consumption needs to decline. Some also claimed reducing their own meat consumption habits.

“I have not reached the point that I do not eat meat at all but somewhere you are part of the problem, so you are also part of the solution.”

(Expert 18)

To summarise, three major themes arise from the literature and interviews, which will be elaborated further in the Section 4. First, the results show the unclarity around the term intensive livestock farming, which can be described as the imprecise use of terminology (Theme 1). Second, as Figure 1 illustrates, there are multiple health hazards and health effects for people living near intensive livestock farms, yet the relationships are not fully understood. For instance, PM is considered a large problem according to the interviewed experts, yet whether PM is the exact cause of respiratory problems remains unclear. Hence, there is knowledge uncertainty about the health effects (Theme 2). Third, experts mention other themes such as animal welfare and loss of biodiversity, suggesting broader value-based concerns (Theme 3).

## 4. Discussion

This study explored the current scientific knowledge, beliefs and concerns about the relationship between intensive livestock farming and human health for residents in the Netherlands. Based on the literature and interviews, the aggregated expert model reinforces the view that intensive livestock farming in the Netherlands is a complex issue, in particular due to imprecise use of terminology, knowledge uncertainty about the health effects, as well as broader value-based concerns.

From the interviews, there is imprecise use of terminology, which “arises because words have different or imprecise meanings” [41]. The standard term to describe the issue in the societal debate is intensive livestock farming (In Dutch: intensieve veehouderij). Intensive livestock farming refers to farms without their own feed production (because of lack of land and consequently animal feed being produced elsewhere) and is limited to specific animals predominantly poultry, dairy goats and pigs. The interviewed experts associated the term predominantly with poultry, pigs and to a lesser extent goat farms, the number of animals, and the scale: i.e., many animals (without quantifying) concentrated on a small surface area.

Experts predominantly use the broader term “livestock farming” when talking about health and intensive livestock farming. Occasionally, they use terms such as “farming (In Dutch: landbouw)”, “intensification”, “scale-enlargement” and “mega farm (In Dutch: megastal)”. In the international literature, other seemingly interchangeable terms are used, such as “industrial food animal production”, “intensive farming”, “(concentrated) animal feeding operations” and “intensive livestock operations” [26,27,28,29,42,43,44,45]. These different ways of describing the issues have different connotations in terms of defining the problem, associations with human health hazards, emphasis on specific value-based concerns and the development of possible solutions.

As interpreted from the expert interviews, the problem from a human health perspective is the high livestock–human density. The term that we think best describes this is “densification” of the livestock industry. As a consequence of this densification, some communities are being exposed to multiple hazards with potential health effects. Also, not all terms may be associated with health hazards relating to animals. For instance, farming includes growing crops besides the rearing of animals. Therefore, it can also refer to health hazards associated with crop production, such as the use of pesticides. The terms intensification, scale enlargement and a mega farm as such do not generate human health problems as the experts repeatedly stated that these are accompanied by investments in technical innovations, such as introducing air-filtering systems. Moreover, using certain terms may result in limiting the problem to specific animals, farm types and sizes. Intensification, scale-enlargement and mega farms are not limited to intensive livestock farms but may also take place in land-based dairy farms as well as biological farms and these also have potential human health hazards [24]. In addition, as mentioned earlier, small farms which are not labelled by the experts as intensive livestock farming yet may be described as livestock farming, may also pose a risk for human health. With respect to broader value-based concerns associated with intensive livestock farming such as animal welfare, experts appear unconvinced that it is limited to intensive livestock farms or mega-farms, as modern stables increasingly take animal welfare into consideration. They talk about animal welfare broadly, in the context of “livestock farming”.

As a result, it appears that the term intensive livestock farming is unclear and does not fully describe the issue. Furthermore, the use of terminology is inconsistent. As a result of this unclarity and inconsistency, the discussion is subject to different interpretations. It is likely that other stakeholders, such as residents and farmers, will also vary in terminology use. Consequently, as words may mean different things to different stakeholders, a “semantic barrier” in communication may emerge as a consequence [46]. There is the risk being caught in what van Eeten describes as a “dialogue of the deaf” [47], a situation between people who talk, but do not listen, causing misunderstanding and disagreements between stakeholders due to different word selection and the interpretations deriving from these words.

The literature shows there are various hazards with potential health effects for people living in close vicinity of livestock farms. However, the literature exemplifies that, with the exception of Q fever and odour, the causes linking the hazards and effects is not fully understood. The most commonly reported health effects of residents living in close proximity of livestock farms in the literature are respiratory system problems, which are associated with many potential hazards, in particular PM. Most experts ranked PM as a large problem, even though there remains a lack of evidence whether PM is the cause for negative respiratory effects among neighbouring residents. Antibiotic-resistant bacteria is considered a broader problem for society but not a specific problem in livestock farming. Zoonotic diseases require continued vigilance and are only considered a potential problem in the case of an outbreak. For example, despite evidence that neighbouring residents have a risk of Q fever infection, experts believe it is currently not a large problem, due to the mandatory measures set in place. Whether the effects from odour should be deemed as a risk for human “health” seems to be controversial. The interviewed experts generally acknowledge it as a large problem for residents, having a significant negative impact on people’s lives. Studies also show that odour may have physical and psychological consequences. Moreover, the threat to health first within the WHO definition of health: “is a state of complete physical, mental and social well-being and not merely the absence of disease or infirmity” [48]. However, the interviewed experts consider it only a minor issue compared to other hazards, possibly because various factors determine the amount of odor and the nuisance experienced such as demographic, socio-economic, personal and cognitive factors [49]. This makes it difficult to establish scientifically-based odor standards. The experts appear to have adopted a more narrow definition of health, namely the absence of disease. Overall, the interviewed experts appear to perceive intensive livestock farming not as a major health problem at least at this moment for neighbouring residents in the Netherlands.

Although the focus of this study was on residential health and intensive livestock farming, it was observed that broader value-based concerns such as (environmental) unsustainability, the loss of biodiversity and landscape aesthetics seemed a more prominent concern among the interviewed experts. In this case, experts voiced their concern on aspects which are beyond their area of expertise and may reflect underlying biospheric values [50]. However, regarding animal welfare, it appears it is something that experts value yet are not necessarily concerned about in the context of intensive livestock farming. In spite of expressing a neutral attitude towards intensive livestock farming, it appears that based on the broader concerns, the experts believe that the livestock system in general in the Netherlands needs transformation.

### 4.1. Strengths and Limitations

This paper provides a comprehensive overview of the hazards and effects for residents living near intensive livestock farms. More importantly, the interviews enabled us to gain insight into whether intensive livestock farming is a problem for residential health in the Netherlands, according to scientific experts by including 20 scientific experts with various backgrounds and expertise on different health hazards in order to identify their pooled knowledge, beliefs and concerns. Despite the combination of open-ended interviews and the diverse sample which facilitated in thoroughly exploring and developing an expert model, these findings cannot be generalised to all scientific experts in the relevant fields, a common limitation of qualitative research. It should also be noted that these findings reflect current expert perceptions. These might be subject to change following future events and scientific knowledge such as a sudden outbreak of a zoonotic disease.

### 4.2. Implications for Future Research

This study discussed the views of merely one, be-it a very influential and often decisive, stakeholder group in the debate about intensive livestock farming: scientific experts. To gain more insight into the other perspectives concerning this issue, it is necessary to explore the mental models of other stakeholders, such as farmers, local residents, interest groups and government officials. Given the knowledge uncertainty about human health and livestock farming, stakeholders may hold different knowledge, beliefs and value-based concerns compared to experts. For instance, stakeholders may disagree with experts that intensive livestock farming is not a major health problem. In an online survey among 1090 Dutch residents by Verhue at al., it was found that people believe that large-scale farms increase the risks of animal husbandry on public health regarding antibiotic-resistant bacteria and infectious diseases [51]. Regarding odour, it is expected that perceptions whether it is a (major) problem will differ as it is a subjective concept; individual-related characteristics may influence how a person perceives odour [52]. Moreover, there is spatial variability of odour concentrations [52]. The extent of serious odor nuisance from livestock farms at the municipality level in the livestock-dense provinces of Noord-Brabant and Limburg ranges between 0% and 9%. However, odour nuisance can strongly vary locally, at the neighbourhood level between 0% to 16% inhabitants experiencing serious odour nuisance [53]. Exploring the broader value-based concerns is also necessary as stakeholders may hold similar but also different concerns. Differences in concerns often may reflect differences in underlying values [50,54]. For instance, Borlée [4] found that neighbouring residents, in contrast to experts, are concerned about antibiotic usage in livestock farms and zoonotic diseases. Our study also illustrates that experts are not necessarily concerned about animal welfare in the context of intensive livestock farming. This may also differ with the perceptions of other stakeholders. Almost four in 10 Dutch people (38%) believe that mega-farms have a negative effect on animal health and welfare [51].

Exploring stakeholders’ perceptions is necessary as scientific experts may rectify stakeholders’ knowledge gaps and prevailing misconceptions regarding intensive livestock farming and human health. However, it will also foster a dialogue, providing opportunities for two-way communication. People can voice their uncertainties and dilemma’s [55], by addressing specific human health concerns or broader concerns other than human health that they find important, but are possibly neglected by experts. Consequently, this influences stakeholders’ reception of the risk communication messages, impacting how people respond to and influence risk management activities as well as policies and laws implemented at municipal, provincial and/or national level, influence consumer behaviour, support for interest groups and political preferences [56].

## 5. Conclusions

Our findings highlight that the terminology used by experts in the debate on intensive livestock farming is unclear, does not fully describe the issue and is used inconsistently. This imprecise terminology is subject to different interpretations and impedes communication between stakeholders. Moreover, despite human health currently being the focal point of discussion in society and in research around intensive livestock farming, experts appear to perceive intensive livestock farming not as a major health problem at least at this moment for neighbouring residents in the Netherlands. As a matter of fact, experts also expressed broader values and concerns. Therefore, our study questions whether dialogues should only focus on residential health or cover broader values and concerns. Perhaps topics other than or in addition to human health need to be addressed in future stakeholder dialogues. To further explore these requirements, we will identify other stakeholders’ (i.e., farmers, neighbouring residents, etc.) knowledge, beliefs and value-based concerns. In addition, recognising the disparities and similarities will be valuable in the light of facilitating constructed dialogues between stakeholders.

## Figures and Tables

**Table 1:** Sample Expert Selection (*n* = 20).

Gender	*n*
Men	12
Women	8
Background	
Public health,	5
Veterinary sciences,	5
Other: engineering, toxicology, molecular biology,	5
Social sciences	5

## Supplementary Materials

The following are available online at https://www.mdpi.com/1660-4601/16/19/3625/s1, Text S1: Interview Questions; Text S2: Results Literature.
