# Supplementary material for: Intensive Livestock Farming and Residential Health: Experts’ Views
Source: Int J Environ Res Public Health. 2019 Sep 27;16(19):3625. doi: 10.3390/ijerph16193625 (PMC6801788; doi:10.3390/ijerph16193625)
Supplement: Supplementary file 1 [file ijerph-16-03625-s001.zip › Supplementary Materials. Interview Questions.pdf]

Supplementary Materials: Interview Questions

Professional background

1. To begin with, can you tell me something about your work and the relationship with intensive livestock farming?

General intensive livestock farming

2. What do you think is "intensive livestock farming"?
    a. What do you understand under "(non-intensive) livestock farming", "mega-farm"?
3. What comes to mind when I say intensive livestock farming?

Public health risks

4. Can you tell me what human health has to do with intensive livestock farming? (negative & positive)?
5. In your opinion, what are the human health risks with regard to intensive livestock farming?
6. Based on the literature, I compiled a list of human health hazards concerning intensive livestock farming (show list) What do you think of this list? Is this complete?

Ranking

7. I have put these human health hazards on cards (show plasticized cards). Now my question is: Which risks do you consider the biggest problem in relation to intensive livestock farming Can you make a top 3 and elaborate your choice?
8. Which risks do you consider the smallest problem in relation to intensive livestock farming? Can you make a top 3 and elaborate your choice?

Specific public health risks

9. When it comes to your own expertise within the context of intensive livestock farming, can you tell me more about _____ (i.e. particulate matter, infectious diseases etc.) and the human health problem?

Other themes

10. In addition to public health, what other issues can you think of when I say in intensive livestock farming?

Concerns

11. As a scientist, are you worried about intensive livestock farming? What are those concerns?

    a. Human health?
    b. Other themes?

12. What do you think citizens are concerned about when it comes to intensive livestock farming? Are there differences between population groups (local residents)?
    a. Human health?
    b. Other themes?

Suggestions other scientific experts

13. Are there scientific experts who you would recommend to interview on this subject?

Before ending the interview, I would like to ask you whether there are still aspects related to intensive livestock farming that have not been covered in this interview but are important for this research?
