# Supplementary material for: Intensive Livestock Farming and Residential Health: Experts’ Views
Source: Int J Environ Res Public Health. 2019 Sep 27;16(19):3625. doi: 10.3390/ijerph16193625 (PMC6801788; doi:10.3390/ijerph16193625)
Supplement: Supplementary file 1 [file ijerph-16-03625-s001.zip › Supplementary Materials. Results Literature.pdf]

Supplementary Materials: Results Literature

Human Health Hazards

PM is the collective name for all microscopic atmospheric particles, which is classified by size: PM10 which are particles smaller than 10 micrometres, PM2.5 and PM1.0. In addition, particulate matter is subdivided into primary and secondary PM [1].

Primary particulate matter is emitted directly into the atmosphere, which include organic material for instance skin flakes, feathers, hair, animal feed, faecal matter [2]. It also consists of microorganisms, a general name for microorganisms such as viruses, bacteria and fungi that are associated with animal production. They can be carried by animals as reservoirs, pathogenic for animals and humans or just present without causing infection or disease. In the model, microorganisms have been subdivided into endotoxins, antimicrobial resistant bacteria, and zoonotic pathogens. Endotoxins are cell-wall components from gram-negative bacteria [3]. Poultry and pig farms are known to emit large quantities of endotoxins. Some bacterial microorganisms are resistant to antimicrobials such as MRSA, Extended Spectrum Beta Lactamases (ESBL producing bacteria) and *Clostridium difficile*. Some microorganisms originating from animals can infect humans. There are many zoonotic pathogens that may be present in livestock and can be transmitted to residents.

Secondary particulate matter is formed from chemical reactions between gases and particles in the atmosphere such as sulphur dioxide, nitrogen oxide and ammonia [4]. Livestock farming contributes to secondary particulate matter by emitting ammonia (NH_3), which is the main precursor of the secondary inorganic aerosols containing ammonium nitrate and ammonia sulphate. NH_3 as well as other compounds such as hydrogen sulphide, volatile organic compounds and particulates from microbial decomposition of manure may produce odour [5].

Nevertheless, it is difficult to distinguish the sources of primary and secondary PM; primary and secondary particulate matter have multiple sources such as industry and traffic, which are known to contribute significantly to the total concentration [6]. In addition, the formation of secondary particulate matter takes time, as emissions of secondary particulate matter spread over large distances.

Manure, which consists of animal faeces used as a fertilizer in agriculture, is considered a reservoir of various hazards in this model. It contributes to (primary) particulate matter; known and unknown microorganisms can originate from animals shedding microorganisms such as zoonotic pathogens, antibiotic resistant bacteria and endotoxins which then spread through the air [7,8]. Zoonotic pathogens found in manure include for instance Salmonella and Campylobacter shed in faeces of infected animals. Also *Coxiella burnetii* found in urine, faeces and birth products may lead to contamination of manure [9]. Also antibiotic resistant bacteria such as MRSA and ESBL might be present in manure. Moreover, manure contains secondary particulate matter such as ammonia, hence

contribute to odour. However, there currently remains a lack of knowledge which point of the role of manure in the health effects and risks of neighbouring residents [8].

Health effects

Various international literature reviews provide contrasting conclusions. Douglas et al [10] state that the association between health outcomes and bioaerosols in nearby communities show mixed findings. Whereas Casey et al [11] conclude that there are consistent and positive associations with living near livestock farms. In contrast, O'Connor et al [12] seem to assert that the associations remain unclear, except for Q fever. All reviews stress the need for more research for stronger evidence. Moreover, despite various studies finding an association with respiratory health, these were performed in different regions predominantly in the vicinity of swine farms, in the Netherlands, USA and Germany. Also, countries differ in farm characteristics and manure management procedures. Therefore, the outcomes of these studies may not be comparable to the Netherlands [13].

Physical and psychological complaints related to odour

Odour can affect negatively affect people physically, psychologically, socially and behaviourally [14]. Physical complaints include increased incidences of headaches, runny, nose, sore throat, excessive coughing, diarrhoea and burning eyes [15]. Psychological complaints include negative mood such as experiencing more tension, depression, anger, fatigue, confusion and less vigor [16,17]. Moreover, odour from hog farms restricts undertaking daily activities and social interactions [18] and reduced quality of life [19]. Similarly, a study in the Netherlands found that a high number of pigs, poultry and cattle in a 500m radius from a home was associated with increased odour annoyance: respiratory (dyspnoea, coughing), gastrointestinal (nausea, upset stomach), neurological (headache, vertigo) and among people with lower back pain stress-related symptoms (fatigue, sleep disturbance) [20].

Respiratory effects

Negative effects including community-acquired pneumonia [21-24], wheezing and reduced lung function [25,26] and COPD exacerbations (chronic bronchitis) [27,28] have been reported. Some studies have found an association with asthma symptoms [29,30] as well as asthma exacerbations [31]. However, these findings are inconsistent [10] as a lower incidence of asthma has also been documented [27,32,33].

The exact nature of the hazards causing these negative as well as positive health effects remain unknown. Various zoonotic pathogens, particulate matter and endotoxins may cause respiratory problems, yet the likelihood is dependent on the livestock type [24]. Exposure to PM is associated with respiratory effects, cardiovascular effects and mortality [34]. In addition, it may aggravate

existing diseases [35]. It is also established that exposure to endotoxins may cause acute respiratory effects and long-term exposure can cause chronic bronchitis and reduced lung function [36]. Yet, knowledge about the health effects in local residents due to exposure to endotoxins from livestock farms is limited [1].

In the Netherlands, spatial associations have been found between living near poultry and goat farms and pneumonia. On average, there are 1650 people with pneumonia per 100,000 inhabitants each year in the research area. Of these, more than 200 lung infections are associated with living in the vicinity of poultry farming or goat farms [37]. Associations were reported between living near (1 km) poultry farms and the incidence of pneumonia between 2009-2014 [6]. A recent study conducted in Pennsylvania also concluded that living near poultry farms may increase the risk of community acquired pneumonia [38]. Experts have speculated that particulate matter and endotoxin missions are the cause, as poultry farms have high emission levels [22-24]. Notably, in 2015 and 2016 the association between living near poultry farms and the incidence of pneumonia in the Netherlands was only weakly present for broilers but no longer statistically significant [39].

Stronger and consistent associations have been found between living near (within 2 km) of goat farms and an increased incidence of pneumonia between the periods 2007-2013 [6] and in the period 2014-2016 [39]. As goat farms emit less particulate matter and endotoxins compared to poultry and pig farms, it is unclear whether the additional pneumonia cases are caused by particulate matter and endotoxins or other unknown zoonotic pathogens [21,23,24].

COPD exacerbations have also been reported in residents living in regions with a high density of livestock farms in the Netherlands [27,28]. It is hypothesised that this could lead to respiratory dysbiosis: “disorder and dysregulation of the microbial ecosystem of the respiratory tract, coupled with a dysregulated host immune response” [27]. On the other hand, in the same regions, there is less asthma (although a positive association was found with asthma and allergic rhinitis among people living within 500 m of a mink farm), allergic rhinitis (nasal allergies, COPD and a lower prevalence of atopy [27,32,33]. It is hypothesised that exposure to diverse microbial component contribute to this inverse association. Yet, the mechanism how microbial diversity may protect against allergies remains to be elucidated.

In addition, temporal associations have been found. An increased concentration of ammonia in the atmosphere originating from livestock farms is correlated with a decrease in lung function. Ammonia is most likely not the primary cause of the effects on the airways but due its conversion into secondary particulate matter [6].

Antimicrobial resistant bacteria carrier/infections

Multiple studies have investigated the prevalence of livestock-associated MRSA among persons not in direct contact with livestock [40-42]. However, due to different research methodologies, the prevalences differ ranging between 0,2% and 0,8%. In the most recent study, the researchers conclude that overall MRSA prevalence among persons without professional contact with livestock living livestock farms is low (0,6%), yet is slightly higher compared to the general population (0,1-0,2%). They conclude that living near farms increases the risk for MRSA carriage. Similarly, according to Casey et al [11], there is sufficient evidence of an association whereas according to O'Connor et al [12] there remains inconsistent evidence. Nonetheless, the exact transmission routes are unknown [42]. No association was found between living near livestock farms and ESBL and Clostridium Difficile carriage [11,43,44].

Zoonotic pathogen carrier/infections

No association was found between Hepatitis E infection/carriage and living in the vicinity of pig farms, despite it being highly prevalent in the pig population in the Netherlands [45]. A recent American study showed that high density poultry farms is associated with campylobacteriosis and infectious diarrhoea in nearby communities [46]. No studies were found examining the relationship between living near livestock farms and the risk for Salmonella infection/carriage. In contrast, *Chlamydia psittaci,* commonly found in parrots and pigeons, have been reported in poultry farms in Belgium and France with occupational risks for human psittacosis infections [47-50]. However, in the Netherlands, *C.gallinaceae* and not *C. psittaci* has been reported in poultry farms [51] and currently there is no clear evidence that there is a risk for people living near poultry farms [52].

Furthermore, little is known about the exposure of inhabitants to avian influenza viruses, which may cause pneumonia [6]. It is expected that the risk for residents to become infected by a LPAI or HPAI avian influenza virus is small as they do not have direct contact with (infected) poultry. A small proportion of the sample neighbouring residents in the South of the Netherlands appear to have antibodies to an avian influenza virus, implying that these people have been exposed to the avian influenza virus. However, a clear relationship with proximity to poultry has not been found [6]. Since the Q fever outbreak in the Netherlands 2007-2010, more is known on the zoonotic pathogenicity of *Coxiella burnetii* for neighbouring residents with strong evidence of a positive association [11,12]. An association with community acquired pneumonia (symptom of Q fever) and living in the vicinity of goat farms has also been found [21] and has been confirmed in a more recent study [53].

References

1. Health, K.P.f.L.F.a.H. *Particulate matter and endotoxins. [in dutch: Fijn stof en endotoxinen]*; Knowledge Platform for Livestock Farming and Human Health: 2017.
2. Winkel, A.; Wouters, I.; Aarnink, A.; Heederik, D.; Ogink, N. *Emissies van endotoxinen uit de veehouderij: Een literatuurstudie voor ontwikkeling van een toetsingskader. [in dutch: Emissions of endotoxins from animal production: A literature survey for development of an assessment framework]*; 1570-8616; Wageningen UR Livestock Research: 2014.
3. Heederik, D.; Sigsgaard, T.; Thorne, P.S.; Kline, J.N.; Avery, R.; Bønløkke, J.H.; Chrischilles, E.A.; Dosman, J.A.; Duchaine, C.; Kirkhorn, S.R. Health effects of airborne exposures from concentrated animal feeding operations. *Environmental health perspectives* **2006**, *115*, 298-302.
4. Cambra-López, M.; Aarnink, A.J.; Zhao, Y.; Calvet, S.; Torres, A.G. Airborne particulate matter from livestock production systems: A review of an air pollution problem. *Environmental pollution* **2010**, *158*, 1-17.
5. Schiffman, S.S.; Walker, J.M.; Dalton, P.; Lorig, T.S.; Raymer, J.H.; Shusterman, D.; Williams, C.M. Potential health effects of odor from animal operations, wastewater treatment, and recycling of byproducts. *Journal of Agromedicine* **2000**, *7*, 7-81.
6. Maassen, K.; Smit, L.; Wouters, I.; van Duijkeren, E.; Janse, I.; Hagenaars, T.; IJzermans, J.; van der Hoek, W.; Heederik, D. *Veehouderij en gezondheid omwonenden*; RIVM rapport: 2016.
7. van Leuken, J.; Hoeksma, P.; Nijsten, D.; Schijven, J.; Schmitt, H.; de Roda Husman, A. *Exploration of the microbiological risks of manure for health: Based on a systematic literature review. [in dutch: Verkenning van de microbiologische risico's van mest voor de gezondheid: Op basis van een systematisch literatuuronderzoek]*; Rijksinstituut voor Volksgezondheid en Milieu: 2017.
8. Health, K.P.f.L.F.a.H. *Manure and manure treatement [in dutch: Mest en mestbewerking]*; Knowledge Platform for Livestock Farming and Human Health: 2019.
9. van den Brom, R.; Roest, H.-J.; de Bruin, A.; Dercksen, D.; Santman-Berends, I.; van der Hoek, W.; Dinkla, A.; Vellema, J.; Vellema, P. A probably minor role for land-applied goat manure in the transmission of coxiella burnetii to humans in the 2007–2010 dutch q fever outbreak. *PLoS One* **2015**, *10*, e0121355.
10. Douglas, P.; Robertson, S.; Gay, R.; Hansell, A.L.; Gant, T.W. A systematic review of the public health risks of bioaerosols from intensive farming. *International journal of hygiene and environmental health* **2018**, *221*, 134-173.
11. Casey, J.A.; Kim, B.F.; Larsen, J.; Price, L.B.; Nachman, K.E. Industrial food animal production and community health. *Current environmental health reports* **2015**, *2*, 259-271.

12. O'Connor, A.M.; Auvermann, B.W.; Dzikamunhenga, R.S.; Glanville, J.M.; Higgins, J.P.; Kirychuk, S.P.; Sargeant, J.M.; Totton, S.C.; Wood, H.; Von Essen, S.G. Updated systematic review: Associations between proximity to animal feeding operations and health of individuals in nearby communities. *Systematic reviews* **2017**, *6*, 86.
13. Borlée, F. Respiratory health effects of livestock farm emissions in neighbouring residents. Utrecht University, the Netherlands, 2018.
14. Blanes-Vidal, V.; Nadimi, E.S.; Ellermann, T.; Andersen, H.V.; Løfstrøm, P. Perceived annoyance from environmental odors and association with atmospheric ammonia levels in non-urban residential communities: A cross-sectional study. *Environmental Health* **2012**, *11*, 27.
15. Wing, S.; Wolf, S. Intensive livestock operations, health, and quality of life among eastern north carolina residents. *Environmental health perspectives* **2000**, *108*, 233-238.
16. Schiffman, S.S.; Miller, E.A.S.; Suggs, M.S.; Graham, B.G. The effect of environmental odors emanating from commercial swine operations on the mood of nearby residents. *Brain research bulletin* **1995**, *37*, 369-375.
17. Horton, R.A.; Wing, S.; Marshall, S.W.; Brownley, K.A. Malodor as a trigger of stress and negative mood in neighbors of industrial hog operations. *American journal of public health* **2009**, *99*, S610-S615.
18. Tajik, M.; Muhammad, N.; Lowman, A.; Thu, K.; Wing, S.; Grant, G. Impact of odor from industrial hog operations on daily living activities. *New Solutions: A Journal of Environmental and Occupational Health Policy* **2008**, *18*, 193-205.
19. Radon, K.; Peters, A.; Praml, G.; Ehrenstein, V.; Schulze, A.; Hehl, O.; Nowak, D. Livestock odours and quality of life of neighbouring residents. *Annals of Agricultural and Environmental Medicine* **2004**, *11*, 59-62.
20. Hooiveld, M.; van Dijk, C.E.; van der Sman-de Beer, F.; Smit, L.A.; Vogelaar, M.; Wouters, I.M.; Heederik, D.J.; Yzermans, C.J. Odour annoyance in the neighbourhood of livestock farming–perceived health and health care seeking behaviour. *Annals of Agricultural and Environmental Medicine* **2015**, *22*.
21. Smit, L.A.; van der Sman-de Beer, F.; Opstal-van Winden, A.W.; Hooiveld, M.; Beekhuizen, J.; Wouters, I.M.; Yzermans, J.; Heederik, D. Q fever and pneumonia in an area with a high livestock density: A large population-based study. *PloS one* **2012**, *7*, e38843.
22. Smit, L.A.; Boender, G.J.; de Steenhuijsen Piters, W.A.; Hagenaars, T.J.; Huijskens, E.G.; Rossen, J.W.; Koopmans, M.; Nodelijk, G.; Sanders, E.A.; Yzermans, J. Increased risk of pneumonia in residents living near poultry farms: Does the upper respiratory tract microbiota play a role? *Pneumonia* **2017**, *9*, 3.
23. Freidl, G.S.; Spruijt, I.T.; Borlée, F.; Smit, L.A.; van Gageldonk-Lafeber, A.B.; Heederik, D.J.; Yzermans, J.; van Dijk, C.E.; Maassen, C.B.; van der Hoek, W. Livestock-associated

risk factors for pneumonia in an area of intensive animal farming in the netherlands. *PloS one* **2017**, *12*, e0174796.

24. Kalkowska, D.A.; Boender, G.J.; Smit, L.A.; Baliatsas, C.; Yzermans, J.; Heederik, D.J.; Hagenaars, T.J. Associations between pneumonia and residential distance to livestock farms over a five-year period in a large population-based study. *PloS one* **2018**, *13*, e0200813.
25. Radon, K.; Schulze, A.; Ehrenstein, V.; van Strien, R.T.; Praml, G.; Nowak, D. Environmental exposure to confined animal feeding operations and respiratory health of neighboring residents. *Epidemiology* **2007**, *18*, 300-308.
26. Schinasi, L.; Horton, R.A.; Guidry, V.T.; Wing, S.; Marshall, S.W.; Morland, K.B. Air pollution, lung function, and physical symptoms in communities near concentrated swine feeding operations. *Epidemiology (Cambridge, Mass.)* **2011**, *22*, 208.
27. Borlée, F.; Yzermans, C.J.; van Dijk, C.E.; Heederik, D.; Smit, L.A. Increased respiratory symptoms in copd patients living in the vicinity of livestock farms. *European Respiratory Journal* **2015**, *46*, 1605-1614.
28. van Dijk, C.E.; Garcia-Aymerich, J.; Carsin, A.-E.; Smit, L.A.; Borlée, F.; Heederik, D.J.; Donker, G.A.; Yzermans, C.J.; Zock, J.-P. Risk of exacerbations in copd and asthma patients living in the neighbourhood of livestock farms: Observational study using longitudinal data. *International journal of hygiene and environmental health* **2016**, *219*, 278-287.
29. Mirabelli, M.C.; Wing, S.; Marshall, S.W.; Wilcosky, T.C. Asthma symptoms among adolescents who attend public schools that are located near confined swine feeding operations. *Pediatrics* **2006**, *118*, e66.
30. Sigurdarson, S.T.; Kline, J.N. School proximity to concentrated animal feeding operations and prevalence of asthma in students. *Chest* **2006**, *129*, 1486-1491.
31. Rasmussen, S.; Casey, J.; Bandeen-Roche, K.; Schwartz, B. Proximity to industrial food animal production and asthma exacerbations in pennsylvania, 2005–2012. *International journal of environmental research and public health* **2017**, *14*, 362.
32. Borlée, F.; Yzermans, C.J.; Krop, E.J.; Maassen, C.B.; Schellevis, F.G.; Heederik, D.J.; Smit, L.A. Residential proximity to livestock farms is associated with a lower prevalence of atopy. *Occup Environ Med* **2018**, *75*, 453-460.
33. Smit, L.A.; Hooiveld, M.; van der Sman-de Beer, F.; Opstal-van Winden, A.W.; Beekhuizen, J.; Wouters, I.M.; Yzermans, C.J.; Heederik, D. Air pollution from livestock farms, and asthma, allergic rhinitis and copd among neighbouring residents. *Occup Environ Med* **2014**, *71*, 134-140.
34. Ross, M.A. Integrated science assessment for particulate matter. *US Environmental Protection Agency: Washington DC, USA* **2009**, 61-161.
35. (RIVM), R.v.V.e.M. *Dossier "particulate matter". [in dutch: Dossier: "Fijn stof"]*; RIVM, 2013.

36. Netherlands, H.C.o.t. Endotoxins. Health-based recommended occupational exposure limit. Health Council of the Netherlands: The Hague, 2010; Vol. 2010/04OSH.
37. RIVM. Pneumonia occurs more often near livestock farms. https://www.rivm.nl/en/news/pneumonia-occurs-more-often-near-livestock-farms
38. Poulsen, M.N.; Pollak, J.; Sills, D.L.; Casey, J.A.; Nachman, K.E.; Cosgrove, S.E.; Stewart, D.; Schwartz, B.S. High-density poultry operations and community-acquired pneumonia in pennsylvania. *Environmental Epidemiology* **2018**, *2*, e013.
39. IJzermans, C.; Smit, L.; Heederik, D.; Hagenaars, T.; Baliatsas, C.; Dückers, M.; Huss, A.; Hogerwerf, L.; Post, P.; Boender, G. *Livestock farming and health of local residents iii: Pneumonia in the vicinity of goat and poultry farms; updating data from general practices 2014-2016. [in dutch: Veehouderij en gezondheid omwonenden iii: Longontsteking in de nabijheid van geiten-en pluimveehouderijen; actualisering van gegevens uit huisartspraktijken 2014-2016]*; 9461225156; Nivel: 2018.
40. Van Cleef, B.A.; Verkade, E.J.; Wulf, M.W.; Buiting, A.G.; Voss, A.; Huijsdens, X.W.; van Pelt, W.; Mulders, M.N.; Kluytmans, J.A. Prevalence of livestock-associated mrsa in communities with high pig-densities in the netherlands. *PloS one* **2010**, *5*, e9385.
41. Paget, J.; Aangenend, H.; Kühn, M.; Hautvast, J.; van Oorschot, D.; Loohuis, A.O.; van der Velden, K.; Friedrich, A.W.; Voss, A.; Köck, R. Mrsa carriage in community outpatients: A cross-sectional prevalence study in a high-density livestock farming area along the dutch-german border. *PloS one* **2015**, *10*, e0139589.
42. Zomer, T.P.; Wielders, C.C.; Veenman, C.; Hengeveld, P.; van der Hoek, W.; de Greeff, S.C.; Smit, L.A.; Heederik, D.J.; Yzermans, C.J.; Bosch, T. Mrsa in persons not living or working on a farm in a livestock-dense area: Prevalence and risk factors. *Journal of Antimicrobial Chemotherapy* **2017**, *72*, 893-899.
43. Huijbers, P.M.; de Kraker, M.; Graat, E.A.; van Hoek, A.H.; van Santen, M.G.; de Jong, M.C.; van Duijkeren, E.; de Greeff, S.C. Prevalence of extended-spectrum β-lactamase-producing enterobacteriaceae in humans living in municipalities with high and low broiler density. *Clinical Microbiology and Infection* **2013**, *19*, E256-E259.
44. Zomer, T.P.; van Duijkeren, E.; Wielders, C.; Veenman, C.; Hengeveld, P.; Van Der Hoek, W.; De Greeff, S.; Smit, L.; Heederik, D.; Yzermans, C. Prevalence and risk factors for colonization of clostridium difficile among adults living near livestock farms in the netherlands. *Epidemiology & Infection* **2017**, *145*, 2745-2749.
45. van Gageldonk-Lafeber, A.B.; van der Hoek, W.; Borlée, F.; Heederik, D.J.; Mooi, S.H.; Maassen, C.B.; Yzermans, C.J.; Rockx, B.; Smit, L.A.; Reimerink, J.H. Hepatitis e virus seroprevalence among the general population in a livestock-dense area in the netherlands: A cross-sectional population-based serological survey. *BMC infectious diseases* **2017**, *17*, 21.

46. Poulsen, M.N.; Pollak, J.; Sills, D.L.; Casey, J.A.; Rasmussen, S.G.; Nachman, K.E.; Cosgrove, S.E.; Stewart, D.; Schwartz, B.S. Residential proximity to high-density poultry operations associated with campylobacteriosis and infectious diarrhea. *International journal of hygiene and environmental health* **2018**, *221*, 323-333.
47. Laroucau, K.; De Barbeyrac, B.; Vorimore, F.; Clerc, M.; Bertin, C.; Harkinezhad, T.; Verminnen, K.; Obeniche, F.; Capek, I.; Bébéar, C. Chlamydial infections in duck farms associated with human cases of psittacosis in france. *Veterinary microbiology* **2009**, *135*, 82-89.
48. Dickx, V.; Vanrompay, D. Zoonotic transmission of chlamydia psittaci in a chicken and turkey hatchery. *Journal of medical microbiology* **2011**, *60*, 775-779.
49. Lagae, S.; Kalmar, I.; Laroucau, K.; Vorimore, F.; Vanrompay, D. Emerging chlamydia psittaci infections in chickens and examination of transmission to humans. *Journal of medical microbiology* **2014**, *63*, 399-407.
50. Laroucau, K.; Aaziz, R.; Meurice, L.; Servas, V.; Chossat, I.; Royer, H.; De Barbeyrac, B.; Vaillant, V.; Moyen, J.; Meziani, F. Outbreak of psittacosis in a group of women exposed to chlamydia psittaci-infected chickens. *Eurosurveillance* **2015**, *20*, 21155.
51. Heijne, M.; Hogerwerf, L.; Dijkstra, F.; van der Goot, J.; Heddema, E.; Kroneman, A.; Notermans, D.; Pannekoek, Y.; de Rosa, M.; te Wierik, M. One health collaboration in the approach to psittacosis. [in dutch: One health-samenwerking in de aanpak van psittacose]. *MEDISCHE MICROBIOLOGIE* **2017**, 43.
52. Hogerwerf, L.; Holstege, M.M.; Benincà, E.; Dijkstra, F.; van der Hoek, W. Temporal and spatial analysis of psittacosis in association with poultry farming in the netherlands, 2000–2015. *BMC infectious diseases* **2017**, *17*, 519.
53. Huijskens, E.; Smit, L.; Rossen, J.; Heederik, D.; Koopmans, M. Evaluation of patients with community-acquired pneumonia caused by zoonotic pathogens in an area with a high density of animal farms. *Zoonoses and public health* **2016**, *63*, 160-166.
